# Neck swelling from a retropharyngeal abscess caused by penicillin-resistant *Streptococcus pneumoniae*: a case report

**DOI:** 10.1186/1756-0500-7-291

**Published:** 2014-05-10

**Authors:** Yukiyoshi Hyo, Hisaki Fukushima, Tamotsu Harada

**Affiliations:** 1 Department of Otolaryngology, Kawasaki Medical School, 577 Matsushima, Kurashiki, Okayama 701-0192, Japan

**Keywords:** Retropharyngeal abscess, Cystic lymphangioma, Penicillin-resistant *Streptococcus pneumoniae*

## Abstract

**Background:**

In small children, retropharyngeal abscesses usually occur after upper respiratory tract infections. Unlike in adults, these abscesses are difficult to diagnose in small children, and can rapidly develop into deep neck or mediastinal abscesses.

**Case presentation:**

A 2-year-old Japanese boy recently presented to our department with a chief complaint of neck swelling. Physical examination revealed bilateral tonsillitis and swelling of the left posterior pharyngeal wall. Emergency neck computed tomography angiography showed a contrast-enhanced abscess cavity posterior to the left retropharyngeal space, and a low-density area surrounded by an area without contrast enhancement in the posterior neck. The latter was suspected to be a deep neck infection secondary to a retropharyngeal abscess. After surgery, the patient was diagnosed with a retropharyngeal abscess and concurrent cystic lymphangioma. The lesions improved after intraoral incision and drainage, and administration of antibiotics.

**Conclusion:**

Lymphangiomas and retropharyngeal abscesses are both known to be more common in children than in adults. However, we found no other reports of concomitant presentation of lymphangioma and retropharyngeal abscess in the literature.

## Background

Retropharyngeal abscesses occur more often in children than in adults, and are not rare [1]. Although the first-line treatment is usually surgical incision and drainage [2], a few recent case studies reported successful treatment with antimicrobial agents only [3]. Retropharyngeal abscesses have been reported to cause deep neck and mediastinal abscesses in some cases. In children, the symptoms of retropharyngeal abscesses are less specific than in adults, and physical examination is less likely to reveal definitive findings, which can cause delayed diagnosis. We recently encountered a patient who presented with a chief complaint of neck swelling, and was subsequently found to have a retropharyngeal abscess caused by drug-resistant pneumococcal infection, with a concurrent cervical tumour. We report here our surgical and medical treatment of concomitant retropharyngeal abscess and cystic lymphangioma, and review the relevant literature.

## Case presentation

**Figure 1:**
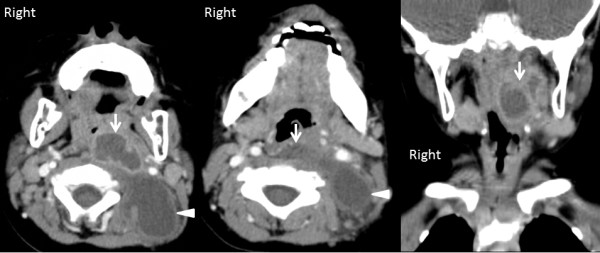
**Neck computed tomography angiography findings.** There was a low-density cavity surrounded by a high-density area (arrow) in the retropharyngeal space. A poorly enhanced low-density area was also observed in the posterior neck (arrowhead).

**Figure 2:**
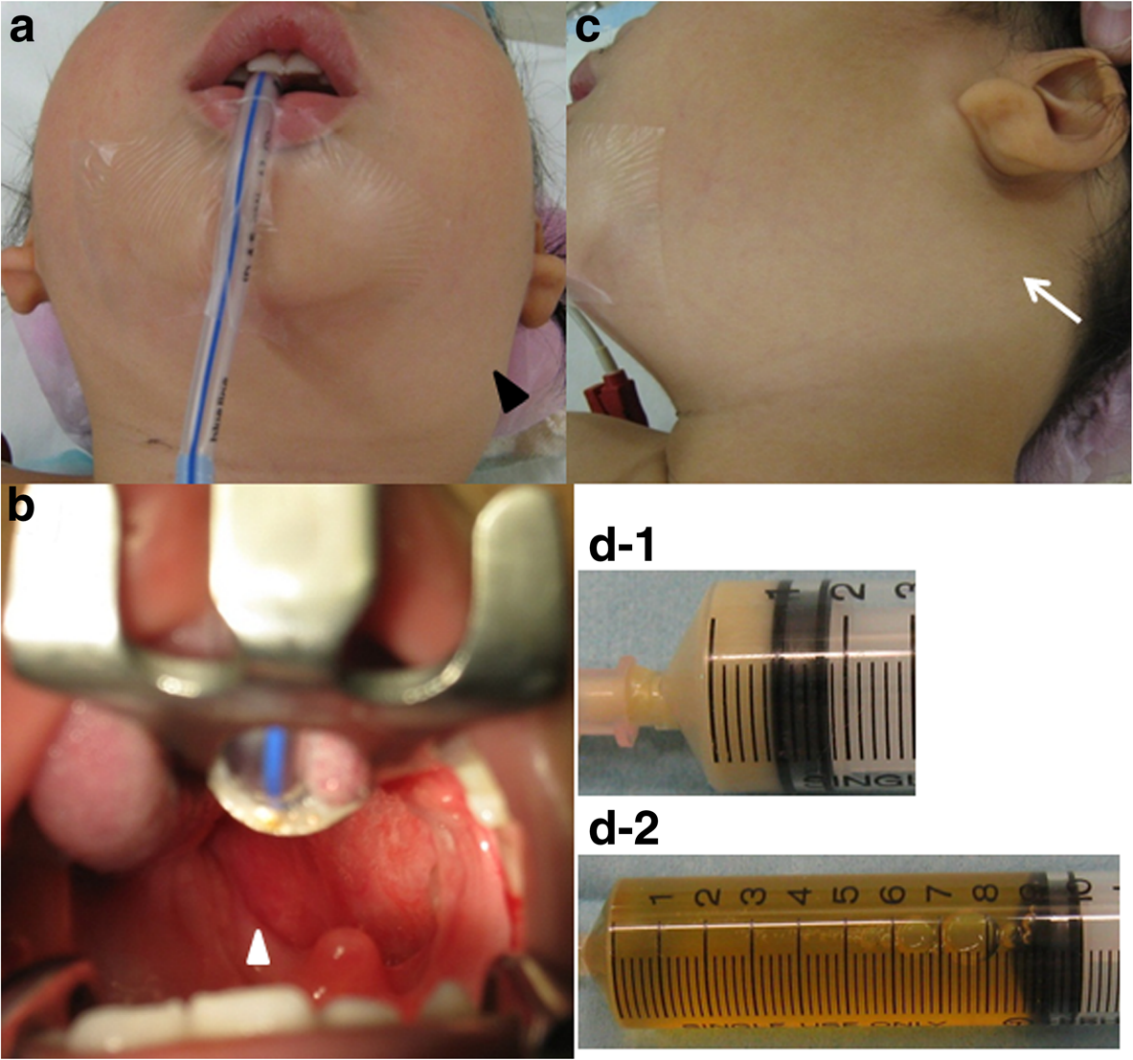
**Operative findings. (a)** Operative view of the posterior neck swelling. **(b)** Operative view of the oral cavity, showing swelling of the left posterior pharyngeal wall. **(c)** Operative view of the posterior neck swelling. **(d-1)** Purulent fluid (1 mL) was aspirated from the left pharyngeal wall swelling by needle puncture. **(d-2)** Fluid aspirated from the posterior neck mass.

**Figure 3:**
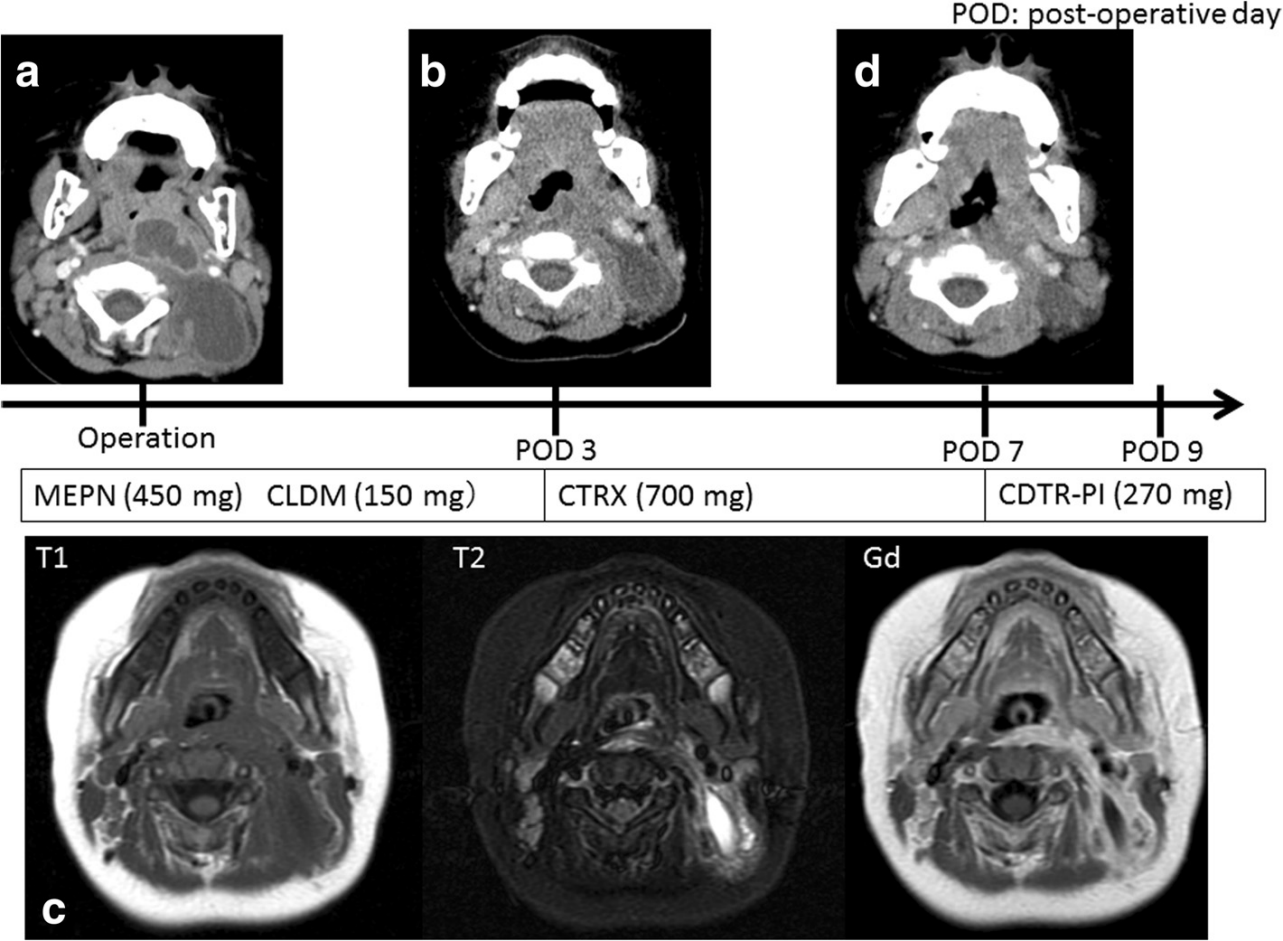
**Postoperative progress. (a)** Preoperative neck CT angiography image. **(b)** CT image on postoperative day (POD) 3, showing improvement of the retropharyngeal abscess but recurrence of the posterior neck swelling. **(c)** MRI on POD 3, showing a lesion with low signal intensity on T1-weighted images and high signal intensity on T2-weighted images, and an area of low signal intensity surrounded by an area of high signal intensity on post-gadolinium images. **(d)** CT image on POD 7, showing slight asymmetry between the right and left sides of the neck. MEPN, meropenem; CLDM, clindamycin; CTRX, ceftriaxone; CDTR-PI, cefditoren-pivoxil.

A 2-year-old Japanese boy was hospitalised with a 2-day history of fever and neck swelling. He had visited a nearby hospital the previous day and had been prescribed clarithromycin. However, the neck swelling increased rapidly, and he was referred to our hospital. On admission, his temperature was 37.5°C and blood pressure was 118/80 mmHg. Physical examination revealed bilateral tonsillitis and swelling of the left posterior pharyngeal wall, and a mildly tender soft mass on his left posterior neck. Laboratory tests revealed a white blood cell count of 14,840 cells/μL and C-reactive protein level of 1.70 mg/dL. Emergency neck computed tomography (CT) angiography showed an enhanced abscess cavity posterior to the left retropharyngeal space, and a low-density area surrounded by an area without contrast enhancement in the posterior neck (Figure 1). The latter was suspected to be a deep neck infection secondary to the retropharyngeal abscess. Based on these findings, we performed surgical incision and drainage under general anaesthesia. After induction of anaesthesia, we used a gag to hold the mouth open, and examined the patient’s oral cavity to locate the exact site of swelling in the left posterior pharyngeal wall (Figure 2). After aspirating 1 mL of purulent fluid by needle puncture, we made an incision over the abscess and washed the abscess cavity with physiological saline. Culture of the abscess fluid was positive for penicillin-resistant *Streptococcus pneumoniae* (PRSP). We also percutaneously punctured the mass on the posterior neck, and drained approximately 10 mL of clear yellowish lymph, but no pus. Rapid cytology showed that this fluid contained mostly protein-like substances and lymphocytes. The mass was no longer palpable after drainage, and no further surgery was performed. The patient was placed under observation with postoperative administration of meropenem (450 mg) and clindamycin (150 mg). On postoperative day 3, his laboratory test results showed improvement, and the antibiotic therapy was changed to ceftriaxone sodium hydrate (700 mg) based on the sensitivities of the cultured organisms. There were signs of improvement in the pharynx, but the mass on the posterior neck had recurred. Repeat neck CT angiography showed a low-density area in the posterior neck, and signs of improvement in the posterior pharyngeal wall. Subsequent magnetic resonance imaging (MRI) angiography showed an area with low signal intensity on T1-weighted images and high signal intensity on T2-weighted images, and an area of low signal intensity surrounded by an area of high signal intensity on post-gadolinium images (Figure 3). The lesion in the posterior neck was diagnosed as a cystic lymphangioma based on the MRI findings and the results of the needle-puncture biopsy culture. Although repeat CT on postoperative day 7 showed slight asymmetry between the left and right retropharyngeal spaces, the patient was discharged as there was no observable asymmetry of the posterior pharyngeal wall, and the posterior neck mass continued to decrease in size. He was followed up at our outpatient clinic for a year, and there was no recurrence of the lymphangioma during that time.

## Discussion

Retropharyngeal infection most commonly occurs in children aged younger than 6 years. Early detection of a developing retropharyngeal abscess can alleviate the need for surgical drainage. Infections originate in the lymphatic chains that drain the upper airway and pharynx. By 3 to 4 years of age, the retropharyngeal lymph node system begins to atrophy, and the risks of inflammation and infection subsequently decrease [4].

Although retropharyngeal abscesses have few specific symptoms, 50% of patients experience fever, stiff neck, neck pain, and difficulty swallowing [5]. However, retropharyngeal abscesses often occur after upper respiratory tract infections, and these symptoms are not specific to the development of an abscess. Previous studies reported that the most common symptoms at presentation were decreased oral intake (92%), neck pain (89%), and neck swelling or mass (79–83%) [6]. Although it is not uncommon for patients with retropharyngeal abscesses to present with a main complaint of neck swelling, this is often associated with enlarged lymph nodes or a deep neck abscess, and none of the previously reported cases had a cystic lymphangioma.

CT angiography is an effective diagnostic imaging modality for the detection of retropharyngeal abscesses [7]. Contrast-enhanced CT is the radiological modality of choice for evaluating retropharyngeal abscesses, and is highly sensitive but not very specific. MRI is better than CT for imaging soft tissue masses such as cystic lymphangiomas. We believe that CT angiography is also useful for the investigation of retropharyngeal abscesses.

Because retropharyngeal abscesses are often accompanied by upper respiratory tract infections, the microorganisms isolated from these abscesses are often similar to those isolated from patients with upper respiratory tract infections. In a study by Hoffmann *et al*., [8] the bacteria isolated from retropharyngeal abscesses included Streptococcus spp. (72%), *S. pyogenes* (41%), *Staphylococcus aureus* (13%), *Candida* (6%), and *Haemophilus influenzae* (3%). The proportion of cases with *S. aureus* infection was particularly high in children aged less than 1 year. The rates of detection of methicillin-resistant *S. aureus* are increasing. Many patients have also been shown to have mixed infections. Brook [9] performed needle aspirations on 14 paediatric patients with retropharyngeal abscesses and found that 12 had mixed anaerobic and aerobic infections, including Streptococcus spp., *S. aureus*, and *H. influenzae*. Studies by Asmar [10] and Craig *et al.*[11] had similar findings. However, antibiotic-resistant bacteria were rarely isolated in these studies, suggesting that isolation of PRSP in the current patient can be attributed to the high prevalence of antibiotic-resistant bacteria in Japan [12].

Lymphangiomas are uncommon lesions of the lymphatic channels, and are often present at birth and usually diagnosed during childhood, mostly before the age of 2 years. Diagnosis is by MRI and fine-needle aspiration cytology findings [13]. In the past, surgery was the treatment of choice for lymphangiomas. However, because of surgical complications including nerve injury, cyst recurrence, and cosmetic problems, OK-432 therapy has recently become the treatment of choice. Injection of OK-432 into the cyst produces inflammation [14,15], and induction of cytokines in the cells of the cyst wall results in fibrotic adhesions in the cyst with resolution of fluid accumulation. In the present case, we hypothesize that inflammation arising from the retropharyngeal abscess also caused inflammation of the congenital lymphangioma. The cystic lymphangioma then remained inflamed after resolution of the retropharyngeal abscess, but resolved after OK-432 therapy.

Lymphangiomas and retropharyngeal abscesses are both known to be more common in children than in adults. However, we found no other reports of concomitant presentation of lymphangioma and retropharyngeal abscess in the literature. Concomitant occurrence of these conditions may increase with increasing age.

## Conclusion

We reported here a case of retropharyngeal abscess caused by PRSP in a young child who presented with neck swelling. Investigation revealed a retropharyngeal abscess and an enlarged cystic lymphangioma secondary to the abscess. After intraoral surgical incision and drainage and administration of antibiotics, the patient recovered uneventfully.

## Consent

Written informed consent was obtained from the patient’s parents for publication of this case report and any accompanying images. A copy of the written consent is available for review by the Editor-in-Chief of this journal.

## Competing interests

The authors declare that they have no competing interests.

## Authors’ contributions

YH and HF were the main consultant surgeons involved in the management of the patient. YH reviewed the literature and wrote the manuscript. TH was involved in caring for the patient in hospital and contributed the case history notes used in this report. All authors read and approved the final manuscript.
